# Estrogen receptor coregulators and pioneer factors: the orchestrators of mammary gland cell fate and development

**DOI:** 10.3389/fcell.2014.00034

**Published:** 2014-08-12

**Authors:** Bramanandam Manavathi, Venkata S. K. Samanthapudi, Vijay Narasimha Reddy Gajulapalli

**Affiliations:** Department of Biochemistry, School of Life Sciences, University of HyderabadHyderabad, India

**Keywords:** estrogen, estrogen receptor, coregulators, pioneer factors, mammary gland development

## Abstract

The steroid hormone, 17β-estradiol (E2), plays critical role in various cellular processes such as cell proliferation, differentiation, migration and apoptosis, and is essential for reproduction and mammary gland development. E2 actions are mediated by two classical nuclear hormone receptors, estrogen receptor α and β (ERs). The activity of ERs depends on the coordinated activity of ligand binding, post-translational modifications (PTMs), and importantly the interaction with their partner proteins called “coregulators.” Because coregulators are proved to be crucial for ER transcriptional activity, and majority of breast cancers are ERα positive, an increased interest in the field has led to the identification of a large number of coregulators. In the last decade, gene knockout studies using mouse models provided impetus to our further understanding of the role of these coregulators in mammary gland development. Several coregulators appear to be critical for terminal end bud (TEB) formation, ductal branching and alveologenesis during mammary gland development. The emerging studies support that, coregulators along with the other ER partner proteins called “pioneer factors” together contribute significantly to E2 signaling and mammary cell fate. This review discusses emerging themes in coregulator and pioneer factor mediated action on ER functions, in particular their role in mammary gland cell fate and development.

## Introduction

Mammary gland development occurs postnatally unlike other human organs (Russo and Russo, 2004). The ovarian hormones, 17β-estradiol (hereafter referred to as E2) and progesterone play a pivotal role in mammary gland development. Although prenatal development of mammary gland is relatively independent of these steroid hormones, pronounced growth occurs during puberty which requires E2. Hence, the hormone-dependent mammary gland development occurs only after puberty. The ovarian hormones impact profound morphogenetic changes in the development of gland by inducing ductal elongation, side branching, terminal end bud (TEB) formation and alveologenesis (Brisken and O'malley, 2010).

E2 exerts its biological functions through specific ligand-inducible nuclear receptors, namely estrogen receptors (ERs) ERα and ERβ. These receptors are encoded by genes located on two different chromosomes and share considerable sequence homology (Nilsson et al., 2001). These proteins regulate the transcription of a diverse array of target genes during development and, in response to specific physiological and pathological signals (Klinge, 2000). Knockout mouse studies have clearly demonstrated that ERα is indispensible for the postnatal development of mammary gland while ERβ is not (Mueller et al., 2002; Mallepell et al., 2006; Feng et al., 2007). The canonical action of the ER involves binding to its ligand with a concomitant dissociation from HSP chaperone proteins, receptor dimerization, nuclear entry and binding to E2 response elements (EREs) located within the promoter/enhancer regions of the target genes to regulate transcription (Klein-Hitpass et al., 1988; Echeverria and Picard, 2010). Accumulating evidence suggests that ERα is preferentially recruited at enhancer regions of target genes upon E2 stimulation (Carroll et al., 2005; Welboren et al., 2009; Gertz et al., 2012; Ross-Innes et al., 2012; Xiao et al., 2012). These enhancer elements modulate target gene expression by forming chromatin loops (Lieberman-Aiden et al., 2009; Sanyal et al., 2012). This is strengthened by the fact that ERα depletion leads to transcriptional repression and loss of chromatin loops, thereby supporting the notion that ERα indeed participates in chromatin loop formation in the breast cancer cells (Fullwood et al., 2009).

In contrast, a recent study suggests that unliganded ERα also binds to large number of chromatin sites in breast cancer cells and, this binding is specifically linked to genes with developmental functions (Caizzi et al., 2014). ERα can also affect gene transcription indirectly through its physical interaction with other transcription factors, such as activator protein 1 (AP1), SP1, nuclear factor-κB (NF-κ B) and E2F1 (Safe, 2001). Functionality of the ERα is further regulated by the post-translational modifications (PTMs) and its cooperative interaction with a special category of proteins called “coregulators,” which exhibit with a coactivator or corepressor activities (Lonard and O'malley, 2007). The importance of coregulators in ER functions as demonstrated *in vitro* generated intense research to decipher their role in mammary gland development. *In vivo* characterization of several coregulators of ERα provides further impetus to their biological roles in normal and pathological conditions.

## The concept of “coregulator”

**Figure 1:**
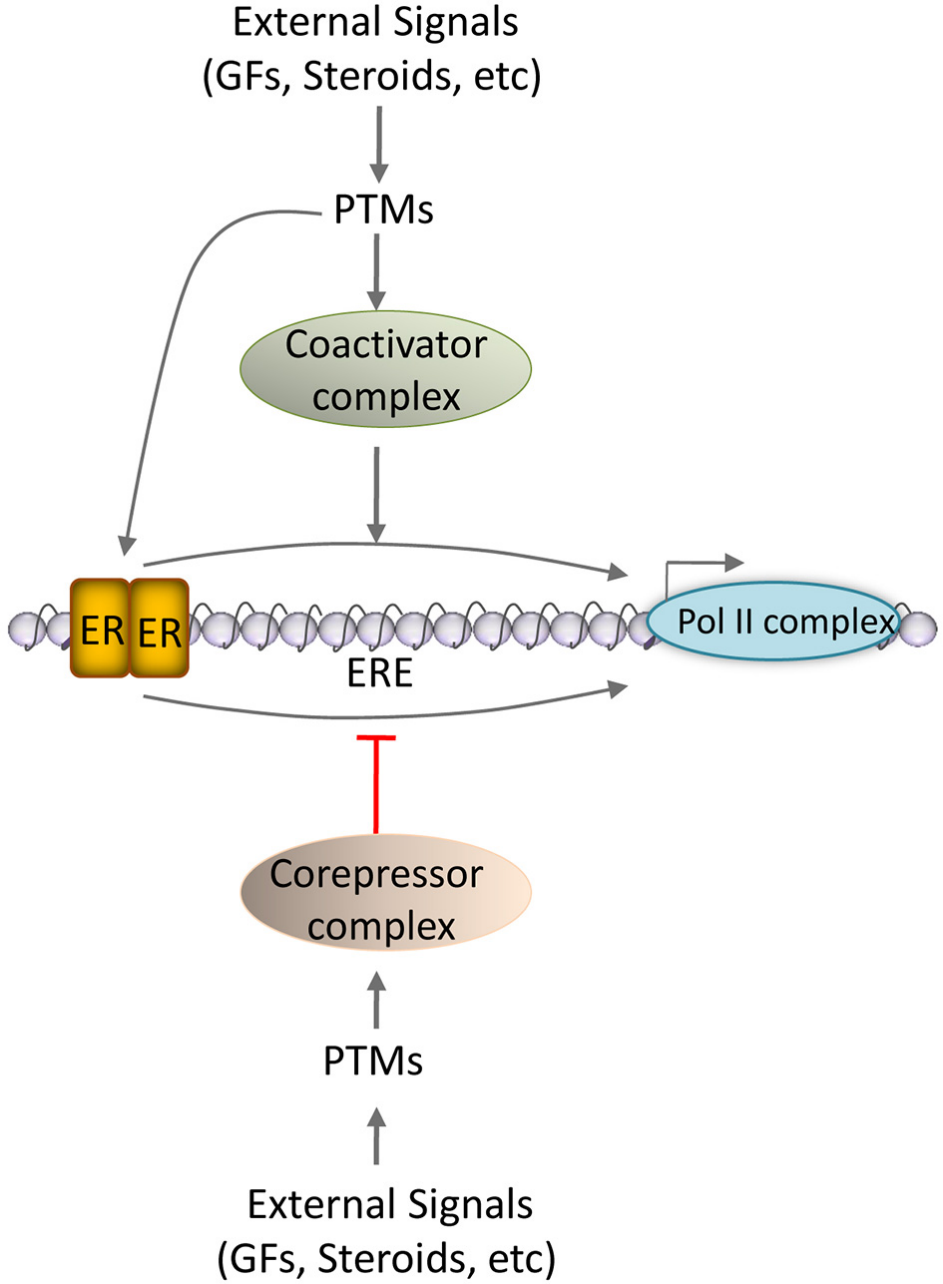
**Coregulator action on ER signaling**. Schematic representation of the action of coregulators on ER activity is comprised of three tiers. In the tier 1, coactivators facilitate ER interaction with RNA Polymerase II complex to enhance the target gene transcription. On the other hand, the corepressors affect ER interaction with RNA Polymerase II complex to suppress the target gene transcription. In tier 2, various post-transcriptional modifications (PTMs) that include phosphorylation, methylation, acetylation, sumoylation, etc., of coregulators further impact on their ability to modulate ER functionality. In tier 3, external signals like growth factors and steroid hormones may influence the post-translational modifications of coregulators. ERE, estrogen response elements.

The concept of coregulator was introduced to the field of nuclear receptors approximately two decades ago (Onate et al., 1995). Though there was confusion initially about classifying these molecules as cofactors based on their similarity to a cofactor of an enzyme, they were later referred as “coregulators” (O'malley and Mckenna, 2008). Unlike the function of a cofactor to an enzyme, coregulator acts as a bridging or helper molecule that helps in forming large protein complexes to modulate appropriate activity on target gene chromatin. Although enzymes exhibit absolute cofactor specificity in a tissue-independent manner, steroid hormone receptors (SHRs) utilizes different tissue-specific coregulators for their activity in a spatio-temporal manner. SHRs being transcription factors, the coregulators may positively or negatively influence the receptor's transcriptional activity by modifying the target gene chromatin. The coregulators that enhance the transcriptional activity of SHRs are called coactivators, and those that decrease it are called corepressors. Though, coregulators form large protein complexes with SHRs, they are just not “bridging” molecules that link SHR and the transcription machinery rather, they often exhibit various enzymatic activities for example, acetylation, methylation, and ubiquitination, that are required for the appropriate regulation of the target gene transcription (Lonard and O'malley, 2007). In general, coactivators contain an intrinsic histone acetyl transferase (HATs) activity. Alternatively they may recruit HATs to the target gene chromatin along with SHRs to enhance the transcriptional activity of the receptor. In contrast, corepressors recruit histone deacetylases (HDACs) to the target gene chromatin to keep the chromatin in closed conformation thus shutting off the target gene transcription (Figure 1). The global actions of coregulators involve chromatin modification and remodeling, initiation of transcription, elongation of RNA chains, mRNA splicing, mRNA translation, miRNA processing, and interestingly degradation of the activated NR-coregulator complexes and termination of the transcriptional response (Lonard and O'malley, 2007). *In vivo* studies using knockout mouse models provided evidence that several of these coregulators are important for E2-dependent mammary growth and the same are discussed here.

## ER coregulators, the orchestrators of E2 signaling

Because majority of breast cancers are ERα positive and coregulators proved to be crucial for ER transcriptional activity, an increased interest in the field has led to the identification of a large number of coregulators (Lonard and O'malley, 2006). Deregulated expression of large number of coregulators is associated with breast cancer development (Manavathi et al., 2013). The first discovered coregulator of ERα is SRC-3/AIB1 (Onate et al., 1995). So far large number of coregulators have been identified for ERα, whereas very few coregulators are known for ERβ (Lonard and O'malley, 2006). Though ERα and ERβ utilize E2 as their physiological ligand, they have overlapping and distinct functions. This could be partly explained by their differential utilization of coregulators. The different classes of ERα (hereafter we referred as ER) coactivators include members of steroid receptor coactivator (SRC)/p160 family, the histone acetyltransferase cAMP responsive element binding protein (CREB)-binding protein (CBP)/p300, ATP-dependent chromatin remodeling complexes like SWI/SNF, E3 ubiquitin-protein ligases and steroid RNA activator (SRA) (Lonard and O'malley, 2006). Majority of coactivators utilize specific motifs called NR boxes or LXXLL motifs (X, any amino acid; L, Leucine) to mediate their interaction with ligand-binding domains of ER (Heery et al., 1997). Conversely, corepressors inhibit ER-mediated gene transcription through a direct interaction with unliganded ER or by utilizing corepressor nuclear receptor (CoRNR) box present in it or by competing with coactivators for ER binding (Hu and Lazar, 1999).

The intriguing question about the ER field is the presence of many coregulators for ER (>200). It is reported that differential expression of coregulators in many tissues account for cell-specific regulation of E2 target gene expression (Misiti et al., 1998; Smith and O'malley, 2004). This implies that the degree of coregulator expression is critical to their ability to influence the transcriptional potential of the ERs that allows fine-tuning of target gene transcription in response to E2 (Brisken and O'malley, 2010). Interestingly, the concentration of several coregulators of ER is subjected to transcriptional regulation by E2, and in turn these coregulators regulate the expression of ERs, thus operating feedback mechanisms that are common in endocrine regulation (Mishra et al., 2004). Various PTMs such as phosphorylation, methylation, ubiquitination, SUMOylation and acetylation also influence the activity of coregulators and, thereby target gene expression (Lonard and O'malley, 2007; O'malley et al., 2008; Han et al., 2009) (Figure 1). Combined or individual modifications on a coregulator can bestow distinct activities for the same coregulator. In this way, a repertoire of coregulators is generated in a cell so that these regulatory molecules are used at appropriate time and conditions.

## Role of ER coregulators in mammary gland development

**Figure 2:**
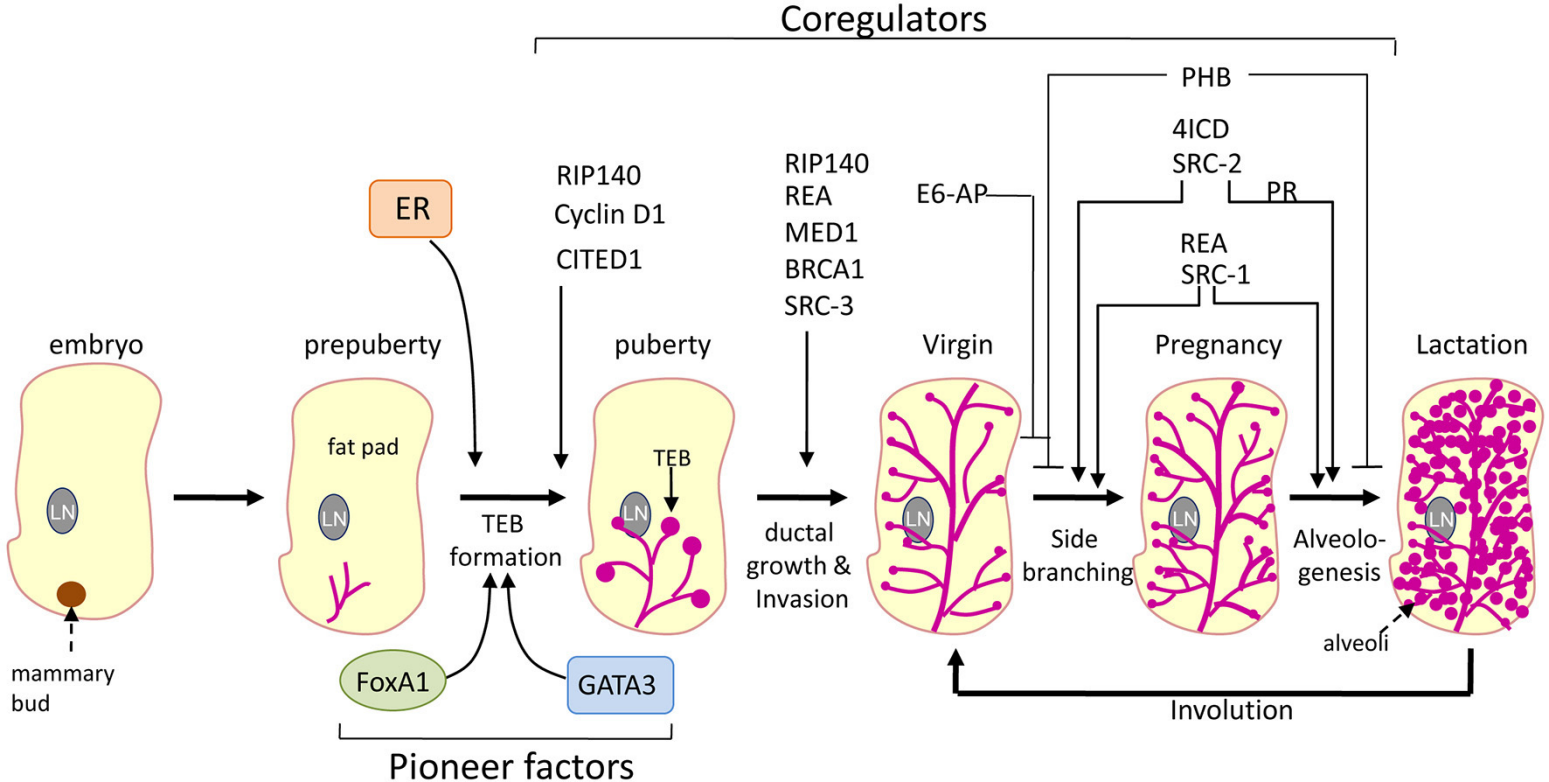
**Role of coregulators and pioneer factors in mammary gland development**. Schematic representation of the role of various coregulators and pioneer factors of ER on different stages of mammary gland development. The thin arrows points to the developmental defects at these stages of mammary gland development due to the loss of the expression of respective coregulator. Whereas the inhibitory lines (-|) points to the suppressive effect of the respective coregulator at the indicated stages of mammary gland development. LN, lymph node; TEB, terminal end buds.

Although the role of E2 in mammary gland development is well established through endocrine disruption procedures, the precise role of its cognate receptor ER in mammary gland development is derived from mouse knockout studies. Disruption of *Esr1* (ER) gene in mouse led to complete abrogation of the mammary gland development indicating that ER is indispensible for the mammary gland development (Mueller et al., 2002; Mallepell et al., 2006; Feng et al., 2007). Because ER-mediated activity depends critically on its coregulator proteins, they are expected to play crucial role in mammary gland development. Knockout approaches were employed to address the role of coregulators in mammary development. Distinct phenotypes such as embryonic lethality, metabolic diseases, impaired reproduction and defects in mammary gland development were evident upon complete ablation of several ER coregulators (Lonard et al., 2007; Dasgupta et al., 2014). The role of various coregulators in mammary gland development is summarized in Figure 2. Mammary gland-specific conditional knockouts are required for understanding the role of coregulators whose complete deletion in mouse results in embryonic lethality. This helps to understand whether they indeed participate in mammary gland development or not. In this review, we will restrict our discussion to the findings till now that connect coregulators to mammary gland development.

## AIB/SRC family

The well characterized amplified in breast cancer (AIB)/steroid receptor coregulator (SRC)/p160 family of coactivators appeared to be involved in regulating uterine growth, embryo implantation, fertility and mammary gland development (Xu et al., 1998, 2000; Gehin et al., 2002; Nishihara et al., 2003). The three SRC's, SRC-1, 2, and 3, have been shown to coactivate ER signaling in mammary epithelial and cancer cells. SRC coactivators contain LXXLL motif which helps them to interact with AF-2 domain of ER and, possesses an intrinsic histone acetyltransferase activity (Mcinerney et al., 1998). The typical mechanism of action by these coregulators includes recruitment of secondary coactivators such as p300, cAMP response element binding protein-binding protein and coactivator-associated arginine methyltransferase 1, which exhibits histone methyltransferase activity, on the target gene promoters to enhance the ER-mediated transcription via chromatin modification (Chen et al., 1999, 2000).

*In vivo* studies established that SRC-1 is required for normal mammary ductal elongation and alveolar development (Xu et al., 1998). Knockout (KO) mouse of *Src-1* had normal body size but showed decreased mammary ductal branching and alveologenesis. Further, *Src-1* KO mice displayed moderate motor dysfunction, delayed development of Purkinje cells, control of energy balance, loss of skeletal response to E2, altered hypothalamic-pituitary-adrenal axis function and hepatic malfunction (Xu et al., 1998; Nishihara et al., 2003). The specific effects on mammary gland in *Src-1* KO mice are that the extent of ductal branching as well as the number of branches was substantially reduced in the mammary glands, spreading of ductal is only half the area of the mammary fat pad and the alveoli that are much less developed in terms of number and size, though milk production is normal. It implies that Src-1 is an important coregulator of ER for ductal branching at puberty and is required for alveologenesis during pregnancy.

Similar to *Src-1* KO mice, the disruption of *Src-2* (GRIP-1 or TIF2) led to severe abnormalities in reproductive function (Gehin et al., 2002). Surprisingly, mammary glands from virgin, pregnant and nursing *Src2* null females were histologically and functionally normal suggesting that *Src-2* is not required for early postnatal development of the mammary gland. Interestingly, SRC-2 protein is expressed in mammary epithelial cells that are positive for progesterone receptor (PgR) indicating that mammary *Src-2* may occupy a crucial role in progesterone-mediated proliferative developments (Mukherjee et al., 2006). Accordingly, *Src-2* KO mice epithelium do not undergo proliferation even in the presence of mammary PgR clearly implying that *Src-2* is indispensable in progesterone-induced mammary ductal side branching and alveologenesis but not for E2 regulated ductal development. Thus, SRC-2 does not integrate into ER signaling *in vivo* directly but could be through PgR signaling.

Similar to the other SRCs, SRC-3 (AIB1, ACTR, and TRAM-1) is also critical for mammary gland development. *Src-3* KO mice display retarded mammary gland development, dwarfism, delayed puberty and reduced female reproductive function (Xu et al., 2000). Tissue expression analysis suggested that *Src-3* is highly expressed in the TEBs and epithelial cells of mammary gland. Despite its expression in TEBs, *Src-3* is not required for TEBs growth. It seems that the retardation of mammary ductal growth in the *Src-3* null mice is due to low levels of E2 (Xu et al., 2000). Therefore, E2 therapy successfully rescued the growth deficiency of mammary ducts suggesting that *Src-3* is not essential for the E2-stimulated ductal growth in virgin mice despite of its high expression in mammary gland epithelial cells. But during alveolar development, *Src-3* is required for progesterone-stimulated cell proliferation and glandular differentiation (Xu et al., 2000). The comparative summary indicates that the physiological role of SRCs is different from one another and proves the diversity among coactivator family members.

## BRCA1

Breast cancer associated protein 1 (BRCA1), a tumor suppressor, was initially identified as a gene predisposed to breast and ovarian cancers (Miki et al., 1994). Rosen group identified BRCA1 as a transcriptional inhibitor of ER (Fan et al., 1999), bringing the first link between ER and BRCA1. BRCA1 inhibits ER transcriptional activity through direct interaction between the BRCA1 and ER proteins and partly, by regulating the expression of p300, a transcriptional coactivator for ER (Fan et al., 2001, 2002). The N-terminal region of BRCA1 interacts with AF-2 domain of ER, while the carboxyl-terminus mediates the repressor function. For instance, BRCA1 inhibits E2-inducible secretion of vascular endothelial growth factor (VEGF) (Zheng et al., 2001; Kawai et al., 2002). Mechanistic studies indicate that BRCA-mediated inhibition of ER transcriptional activity relies on the ability of BRCA1 to mono-ubiquitinate ER which in turn is dependent on the acetylation status of ER (Eakin et al., 2007). Interestingly, BRCA1 is upregulated in response to E2 in mammary epithelial cells and, BRCA1 in turn transcriptionally regulates ER positively by associating with OCT-1, a transcription factor. This implies the existence of a positive feedback mechanism that regulates functional interaction between ER and BRCA1 in breast cancer cells (Spillman and Bowcock, 1996). BRCA1 regulates ER expression positively, hence loss of BRCA1 can lead to ER-negative phenotype providing a molecular basis for the loss of ER expression in the majority of BRCA1 mutant cancers (Hosey et al., 2007).

The physiological link between BRCA1 and mammary gland development was established using knockout mouse models. *Brca1* deletion in mice using tissue-specific conditional knockouts resulted in impaired mammary development (Xu et al., 1999). The *Brca1*^−/−^ mammary glands are smaller due to increased cellular apoptosis and, they also display abnormal ductal morphogenesis. Initial studies suggested an important developmental role for the murine homolog of BRCA1 in the regulation of cell proliferation and differentiation. In consistent with these results, *in vitro* studies using the HC11 murine mammary epithelial cell line showed that *Brca1* mRNA and protein levels increased during lactogenic differentiation (Rajan et al., 1997). Loss of BRCA1 results in impaired differentiation while increased proliferation of MCF10A (a human mammary epithelial cell line), further suggesting its strong association with cellular differentiation. These cellular differentiation functions are probably due to the fact that BRCA1 transcriptionally regulates a number of basal and luminal terminal differentiation markers that define a cell fate within the mammary gland (Hosey et al., 2007; Gorski et al., 2009). Recent studies show that BRCA1 indeed promotes differentiation along the luminal lineage in coordination with Notch signaling (Buckley et al., 2011). Furthermore, BRCA1 is also shown to collaborate with GATA3 to regulate genes crucial for the maintenance of luminal lineage (Kouros-Mehr et al., 2006; Tkocz et al., 2012). It thus implies that BRCA1 is required for luminal cell differentiation during mammary gland development.

## CITED-1

CITED-1, a CBP/p300-binding nuclear protein, functions as a selective coactivator for E2-dependent transcription. In ER-positive breast cancer cells, CITED-1 binds directly to ER through its transactivation domain and sensitizes the cells to E2 by stabilizing the E2-dependent interaction between p300 and ER (Yahata et al., 2001). CITED-1 coactivator function is gene-specific as it doesn't induce ER signature genes such as progesterone receptor, *PgR* or *pS2*, rather it upregulates *TGF-β* in MCF-7 cells (Yahata et al., 2001). Thus the induced TGF-β in turn modulates the proliferative action of E2 (Ewan et al., 2002).

Gene profiling analysis in different regions of mammary ductal tree identified CITED-1 as one of the selectively expressed genes in a subset of luminal and preluminal epithelial cells in the pubertal mammary gland (Howlin et al., 2006). This led to the possibility that CITED-1 may mediate signals in the E2-TGF-β regulatory axis. During puberty, CITED-1 localizes to the luminal epithelial cell population of the mammary ducts and the body cells of the TEBs. Characterization of *Cited-1* KO mice revealed that *Cited-1* is required for ductal growth during puberty as homozygous null mutant's exhibited retarded mammary ductal growth. The other prominent *Cited-1* KO's mammary phenotype was altered ductal morphology with dilated and spatially restricted branches (Howlin et al., 2006). These defects in mammary glands of *Cited-1* KO are attributed to alterations in the transcription of a number of key downstream targets of both E2 and TGF-β. Further, loss of EGFR/ErbB2 ligand (amphiregulin) expression, one of the prime targets of E2, was observed in *Cited-1* null mice. It thus implies the potential maintenance of the ER-CITED-1 co-regulated signaling pathway in the pubertal mammary gland.

## Cyclin D1

Cyclin D1, encoded by *Ccdn1* gene which is located on chromosome *11q13*, forms the regulatory subunit of cyclin-dependent kinases 4 (CDK4) and, was found to be overexpressed in 50% of breast cancers (Ormandy et al., 2003). It is shown to coactivate ER-dependent transcription in a ligand-independent manner (Zwijsen et al., 1998; Lamb et al., 2000). Cyclin D1 competes with BRCA1, which represses ER transactivation, for binding to hinge region of ER thereby it display coactivator function (Wang et al., 2005). In contrast, genome-wide expression profiling in *Ccnd1* KO mice treated with E2 demonstrated that cyclin D1 determines large number of E2-responsive genes *in vivo* (Casimiro et al., 2013). Further, in cyclin D1-silenced (by RNAi) MCF7 cells, it was evident that cyclin D1 is required for E2-mediated gene expression *in vitro*. These results are further substantiated by genome-wide chromatin immunoprecipitation-sequencing (ChIP-Seq) analysis. It was observed that cyclin D1 indeed binds to the E2-ER target gene chromatins, *ErbB3, EphB3*, and their ligands, amphiregulin and matrix metalloproteinase. The above facts suggest that cyclin D1 mediates E2-dependent ER transactivation and is required for convergence of ER and growth factor signaling at a common cis-element of growth factor genes.

Though deletion of *Ccdn1* in mice is not lethal, *Ccdn1* ablation results in the arrest of mammary gland development before lobuloalveolar development, thereby underscoring its definitive role in this process (Fantl et al., 1999). The defects in the mammary gland are due to loss of expression of several E2- and cyclin D1-depenndent genes. In *Ccdn1* KO pregnant mice, the glands do not open due to lack of lobuloalveolar growth. The epithelial cell-specific overexpression of *Ccdn1* in mice leads to mammary carcinoma indicating that the optimal concentration of *Ccdn1* is required for proper development of mammary gland (Wang et al., 1994). This could partly explain why cyclin D1 overexpression is associated with 50% of breast cancers.

## E6-AP

E6-associated protein (E6-AP) is a HECT (homologous to E6-associated protein carboxy-terminal) domain containing E3 ubiquitin ligase that is known to act as a coactivator for ER, PR and RXRα (Nawaz et al., 1999). E6-AP possesses two independent discrete functions: coactivation of ER and ubiquitin-protein ligase activity (Ramamoorthy and Nawaz, 2008). Being a component of the ubiquitin-proteasome pathway, it was presumed that E6-AP may regulate the dynamics of steroid hormone receptor-mediated transcription by modulating the degradation of the transcriptional complexes. However, this still remains elusive as ER stability is known to correlate with E6-AP expression (Gao et al., 2005). Down regulation of E6-AP leads to increased ER levels. On the other hand, E6-AP enhances the transcriptional activity of ER by recruiting chromatin modifiers like p300 at ER target gene promoters (Catoe and Nawaz, 2011). Furthermore, phosphorylation of ER at tyrosine 537 (Y537) by Src kinase is shown to potentiate ER binding to the ubiquitin E6-AP, subsequent ER ubiquitination, target gene activation and ultimately loss of ER function (Sun et al., 2012). This also suggests that a delicate balance between coactivator function and degradation activity of E6-AP, which attributes to its role in mammogenesis as well as breast cancer development.

*E6-ap* KO mice display reduced gonadal size suggesting its role in fertility. Though E6-AP is expressed in mammary gland, E2 and progesterone-stimulated growth of virgin mammary gland was not compromised by *E6-ap* ablation, suggesting that E6-AP is not important in mediating steroid hormone actions *in vivo* (Smith et al., 2002). In another study, it was shown that overexpression of E6-AP (WT) results in impaired mammary gland development. Overexpression of defective E6-AP (C833S) (an ubiquitin ligase-defective), or the loss of *E6-ap* increased lateral branching and alveolus-like protuberances in the mammary gland. The mammary phenotypes observed in the E6-AP transgenic are attributed to the alteration in the level of progesterone receptor-B (*Pgr-B*), an ER target gene (Ramamoorthy et al., 2012). These studies indicate that E6-AP inhibits lateral branching during mammary gland development.

## HER4/4ICD

The HER4/ErbB4 is an EGFR family member that is proteolytically processed at the cell surface to release the cytosolic side fragment i.e., intracellular domain (4ICD), which independently impacts a variety of functions including acting as a coactivator for ER (Naresh et al., 2006; Zhu et al., 2006). Its functions are found to be location specific. For example, nuclear 4ICD in tumor cells acts as a potent ER coactivator leading to E2-stimulated proliferation of breast cancer cells, whereas in mammary gland, it regulates differentiation and lactation via STAT5 pathway (Williams et al., 2004). Cytosolic 4ICD accumulates in mitochondria where it regulates apoptosis (Naresh et al., 2006; Zhu et al., 2006). Endogenous 4ICD interacts with and potentiates ER transactivation in breast cancer cells as loss of 4ICD expression decreases ER-mediated transactivation function, whereas overexpression of 4ICD in the MCF-7 cell line increased E2 stimulation of ERE reporter activity (Zhu et al., 2006). Interestingly, the E2 stimulation of ER drives nuclear translocation of 4ICD. The nuclear localized ER/4ICD complex then selectively is recruited to E2-inducible gene promoters. The 4ICD displays gene-specific coactivation functions toward ER as it coactivates transcription of progesterone receptor (PgR) and stromal cell-derived factor 1 (SDF-1) but not trefoil factor 1 precursor (pS2) (Zhu et al., 2006). Although substantial evidence supports that 4ICD functions as a transcriptional coactivator, the precise mechanism of action toward ER is not known. Interestingly, *Her4* itself is an E2-inducible gene and, ER/4ICD complex recruits to *Her4* promoter in response to E2. Because, Her4 expression is also shown to be required for the growth-promoting action of E2 in breast cancer cells, it provides evidence for the involvement of an autocrine signaling in breast cancer cells. This suggests that 4ICD is a unique coregulator of ER that directly couples extranuclear and nuclear E2 actions in breast cancer cells (Zhu et al., 2006).

The *in vivo* role of 4ICD on ER functions is assessed using knockout mice without *Her4* which is the mature form of 4ICD. *Her-4* KO in mice causes embryonic lethality at day 11 due to defects in heart development (Tidcombe et al., 2003). The rescued *Her4* mutant mice are viable and fertile, but they do not lactate, indicating the role of *Her-4* in mammary gland development. Progesterone receptor (PgR) is known to be required for lobuloalveolar development in mammary gland and, E2 regulates expression of *Pgr-A*, whereas expression of *Pgr-B* is E2 independent (Tanos et al., 2012). Consistent with a role for 4ICD in E2-regulated *PgR* expression *in vitro, PgR-A*, but not *PgR-B*, expression was abolished in mammary glands of *Her4*-null mouse. The study by Risicki et al., provided further evidence that 4ICD is nuclear localized in mammary epithelium and acts as coactivator in the mammary development (Rokicki et al., 2010). In summary, 4ICD is a physiologically important ER coactivator and it cooperates with ER to potentiate *Pgr* expression in the normal breast.

## MED1

MED1, also known as DRP205/TRAP220/PBP, is one of the coactivators of a large ~1.6 MDa protein complex called “Mediator,” which is a part of the RNA polymerase II holoenzyme. “Mediator” bridges the communication between transcriptional activators and RNA polymerase II to influence the transcription (Malik et al., 2004). Therefore, MED1 has been shown to act as a coactivator for ER. The LXXLL motifs of MED1 are crucial for its interactions with nuclear receptors and for nuclear receptor-mediated transcription of target genes (Jiang et al., 2010).

To understand its functional significance in steroid hormone receptor action and mammary gland development, *Med1* LXXLL motif-mutant knockin (KI) mice (*Med1^KI/KI^*) were generated since complete deletion of *Med1* leads to embryonic lethality. *Med1^KI/KI^* mice were fertile and normal but exhibited profound abnormalities at the virgin stage indicating defects in pubertal mammary gland development (Jiang et al., 2010). The defects observed in mammary gland development in *Med1^KI/KI^* mice are attributed to significant impairments both in ER-dependent gene expression in mammary epithelial cells and in E2-stimulated mammary ductal growth further indicating the significance of LXXLL motifs in coactivator function. Because *Med1* gene ablation in mice led to embryonic lethality, a recent study conducted on the *Med1/Med24* double heterozygous KO (dhKO) mice, which are viable, addressed the role of *Med1* in mammary gland development (Hasegawa et al., 2012). Mammary glands from *Med1/Med24* dhKO mice showed profound impediment in ductal branching during puberty, while in single haplo-insufficient mice, glands developed normally. These phenotypic differences point out to the possibility that other subunits of “Mediator” complex are engaged. Further, DNA synthesis of both luminal and basal cells were impaired in double mutant mice. The expression of ER-dependent genes such as *E2F1* and *cyclin D1*, which promote progression through the G(1)/S phase of the cell cycle, was attenuated. These studies established the coactivator functions of “Mediator” complex for ER in normal mammary epithelial cells.

## MTA1 family

The metastasis-associated proteins are a small family of coregulators which comprises of MTA1, MTA2, MTA3, and MTA1s. Individual MTAs takes part as critical components of nuclear remodeling and deacetylation (NuRD) complex, and therefore represses target gene expression through deacetylation of histones in the chromatin (Manavathi and Kumar, 2007). The first target gene identified for MTA1/NuRD complex is ER and the ligand-dependent transactivation functions of ER are repressed in cultured breast cancer cells (Mazumdar et al., 2001). The *Mta1* KO mice are viable but the information about the mammary gland development in these mice is obscure (Manavathi et al., 2007). Mice overexpressing MTA1 (MTA1-Tg) under the control of the mouse mammary tumor virus (MMTV) promoter long terminal repeat was used to understand its role in mammary gland. Extensive side branching and precocious differentiation due to increased proliferation of ductal and alveolar epithelial cells was observed in mammary glands of MTA1-Tg virgin mice (Bagheri-Yarmand et al., 2004). The mammary glands of virgin transgenic mice resemble those from wild-type mice in mid-pregnancy but have shown inappropriate expression of β-casein, cyclin D1 and β-catenin proteins. Interestingly, progesterone receptor-B isoform (*Pgr-B*) expression was down regulated, whereas the progesterone receptor-A (*Pgr-A*) isoform was upregulated in MTA1-Tg mice. Increased expression of Pgr-A resulted in upregulation of PgR target genes such as *Bcl-XL* and *cyclin D1* leading to delayed involution. The results of the study indicated the possible role of MTA1 in mammary gland during branching morphogenesis and alveologenesis.

MTA1s, the short variant of MTA1, has been shown to suppress ER transcription by sequestering the receptor into the cytoplasm (Kumar et al., 2002). MTA1s role in mammary gland development was analyzed by transgenic approach by the same group that generated the MTA1 transgenic mice (Kumar et al., 2010). MTA1s-Tg mammary glands displayed extensive hyperbranching and increased proliferation of ductal and alveolar epithelial cells, which mimicked the phenotypic changes found in the *Wnt*-Tg mice. Overexpression of MTA1s in HC11, a lactogenic differentiation cell line, activated *Wnt1* signaling through MAPK and GSK3-beta pathways, resulting in increased stabilization and nuclear accumulation of β-catenin. Furthermore, mammary glands from the virgin MTA1s-Tg mice showed ductal hyperplasia and ductal carcinoma *in situ*. These findings indicate a possible requirement of MTA1s in mammary gland development during ductal branching and alveologenesis similar to MTA1.

MTA3 also forms a cell type-specific Mi2–NuRD complex. MTA3 has been shown to act as a direct transcriptional repressor of Snail, a master regulator of the epithelial-to-mesenchymal transition (EMT) (Fujita et al., 2003). The role of MTA3 in mammary gland development was explored using a transgenic approach (Zhang et al., 2006). Targeted overexpression of *Mta3* in the mouse mammary gland resulted in a pronounced defect in ductal side branching both in virgin mice and in the early stages of pregnancy, a contrasting phenotype observed in MTA1-Tg mice. Though the mammary glands of both MTA3-Tg mice and wild type mice were similar at early developmental stages, drastic reduction in secondary and tertiary ductal side branching were observed in the Tg mice. This hypobranching phenotype of mammary gland in MTA3 was comparable to the defective branching in *Wnt4* KO mice (Brisken et al., 2000). Further, molecular analysis revealed that the reduced ductal branching is due to suppression of *Wnt4* expression by MTA3-NuRD complex (Zhang et al., 2006). The comparative summary indicates contrasting roles for MTA1 and MTA3 in mammary gland development.

## Prohibitin

Prohibitin (PHB) and REA (Repressor of ER activity), also known as PHB2, belong to a family of proteins that contain an evolutionarily conserved domain, the prohibitin homology domain. PHB plays a diverse role in cellular differentiation, anti-proliferation and morphogenesis (Chowdhury et al., 2014). Overexpression of PHB in breast cancer cells suppresses the ER transcriptional activity, whereas depletion increases the expression of ER target genes (He et al., 2008). PHB interacts with HDAC1 through its coiled domain which partly explains its corepressor activity on ER transactivation functions (He et al., 2008).

The role of PHB in mammary gland development was investigated in *Phb^+/−^* heterozygous mice since complete deletion of *Phb* gene leads to embryonic lethality (He et al., 2008). PHB being a corepressor of ER in cultured cells, it is expected to show a hyper proliferative phenotype in the mammary glands of *Phb^+/−^* mice. Consistent with this notion, *Phb^+/−^* mice displayed increased alveolar lateral budding and ductal side-branching in the hormone-treated mammary gland. The increased level of *cyclin D1*, a direct target of ER, in *Phb^+/−^* mice compared to wild type mice was attributed to this hyper-proliferative state in *Phb^+/−^* mice (He et al., 2008). Accelerated mammary growth in response to steroid hormone treatment was also seen in *Phb^+/−^*mice suggesting an important *in vivo* role for the PHB as a corepressor for ER in controlling steroid-induced mammary morphogenesis.

## REA

Repressor of ER activity (REA) was initially identified as an ER interacting protein using dominant negative ER as bait in two-hybrid screening assays (Montano et al., 1999). REA displays its corepressor functions by enhancing the binding of anti-estrogens such as SERMs to ER. Though REA contains an LXXLL motif near its N-terminus, this motif does not participate in the binding of REA to ER. Rather the C-terminal half of REA interacts with ligand-binding domain of ER. REA functionally competes with coactivators like SRC-1 to modulate ER transcriptional activity which partly explains the corepressor activity of REA on ER-mediated transcription (Delage-Mourroux et al., 2000). Further, it was shown that PTalpha (Prothymosin), another coactivator of ER, antagonizes REA inhibitory action toward ER transcriptional activity by replacing the repressive REA protein away from ER (Martini et al., 2000).

Deletion of both the alleles of REA (PHB2) results in embryonic lethality similar to PHB, whereas heterozygous mice (*Rea^+/−^*) display mammary ductal elongation in virgin animals and, increased lobuloalveolar development during pregnancy followed by delayed mammary gland involution after weaning (Mussi et al., 2006). Since, REA displays corepressor activity on ER, these morphological phenotypes of *Rea^+/−^* mice reflected the increased cell proliferation and ER transcriptional activities. Recently, tissue-specific conditional KO approach was used to investigate the consequences of complete loss of *Rea* in mammary gland (Park et al., 2011). Conditionally deleting one allele or both alleles of the *Rea* gene at different stages of mammary gland development resulted in different mammary phenotypes (Park et al., 2011). During puberty, mice homozygous null for *Rea* in the mammary gland showed severely impaired mammary ductal elongation and morphogenesis, whereas mice heterozygous for *Rea* displayed accelerated mammary ductal elongation and increased numbers of TEBs. In addition, *Rea* mice showed an up-regulation of amphiregulin, the major paracrine mediator of E2-induced ductal morphogenesis. During pregnancy and lactation, mice with homozygous *Rea* gene deletion in mammary epithelium showed a loss of lobuloalveolar structures and increased apoptosis of mammary alveolar epithelium, leading to impaired milk production and significant reduction in growth of their offspring. These studies imply that REA is essential for mammary gland development and, has a gene dosage-dependent role in the regulation of stage-specific physiological functions of the mammary glands (Park et al., 2011).

## RIP140

Receptor-interacting protein 140 (RIP140) was initially identified as an ER interacting factor *in vitro* (Cavailles et al., 1995). Later, it was found to be involved in various regulatory feedback loops and inhibitory cross talks involving several nuclear receptors (White et al., 2004). Though, RIP140 was initially characterized as a nuclear receptor coregulator based on its ability to coactivate steroid hormone receptors, subsequent studies indicated that it can also act as a corepressor (Cavailles et al., 1995; Nautiyal et al., 2013). RIP140 inhibits ER-dependent transcription by competing with coactivators and, also by recruiting histone deacetylases (HDACs) to target gene chromatin (Castet et al., 2004).

RIP140 is expressed in both mammary epithelium and stroma, and its role in the development of mammary gland was assessed using knockout and transgeneic approaches. At puberty, *Rip140* KO mice lacked TEBs at the tips of the ducts, but at maturity (5 months old), in *Rip140* KO mice the mammary tree was relatively less branched as compared to heterozygous and wild type mice (Nautiyal et al., 2013). On the other hand, *Rip140* overexpressing mice exhibited large primary ducts, hyperplasic growth and TEBs. Further, *Rip140*-Tg mice developed larger and denser alveolar structures in mammary epithelium indicating that *Rip140* can influence alveologenesis. The molecular mechanism of the role of *Rip140* in mammary gland development has been attributed to its function as a corepressor that recruited with ER to its target gene promoters including *Areg, Pgr, Ccnd1*, and *Gata3* (Nautiyal et al., 2013). In summary, expression of RIP140 in both mammary epithelial and stromal compartments is required for ductal elongation during puberty and silencing of RIP140 expression leads to a complete loss of the mammary epithelium. These *in vivo* studies thus imply that RIP140 is certainly a coregulator for ER.

## The ER signaling in cell fate decisions

### E2 signaling and ER status in mammary stem cells (MaSC)

Cell fate is simply defined as cellular specification or determination with a unique character and behavior. Tissue transplantation experiments combined with lineage tracing approaches using mouse models provided significant advancement in understanding the mammary cell fate (Stingl, 2009; Van Amerongen et al., 2012). The present understanding suggests that a functional epithelium can be regenerated from mammary stem cells upon transplantation into cleared fat pads. Mammary epithelium contains progenitors that give rise to ductal system and contractile myoepithelium because of their pluripotent capacity (Rosen, 2012). Two main lineages of mammary epithelium, namely luminal epithelial cells and basal myoepithelial cells, generate a functional epithelium. The luminal cells are the ducts that form the milk-secreting cells of the alveoli, whereas the myoepithelial cells that form a layer on the luminal epithelium along the ducts helps in milk secretion during lactation. The luminal cells may be either ER positive (ER+) or ER negative (ER−) but the luminal progenitors are predominantly ER−, with a small number of them being ER^+^ (Regan et al., 2012). The ER+ cells are hormone responsive and support paracrine signaling in luminal epithelium, whereas the ER-luminal cells include the milk secretory cells (Regan et al., 2012). Therefore, all three populations of cells that include myoepithelial, ER+ and ER− luminal cells constitute the differentiated cells of mammary epithelium. Both myoepithelial and luminal lineages can also be derived from the TEBs or TEBs implying that TEBs carry stem cells and progenitor features (Srinivasan et al., 2003). Mammary stem cells require estrogen signaling as ovariectomy in mice markedly diminishes mammary stem cells (MaSC) number and their outgrowth potential *in vivo* indicating a role for E2 in self renewal and maintenance of stemness (Asselin-Labat et al., 2010).

### Pioneer factors in mammary cell fate

In general, the cell fate decisions are driven by tissue-specific transcription factors (TFs) based on the cell fate determination signals. A set of transcription factors forming a genetic network with a defined regulatory system controls cell fate determination and subsequently organ development. A well orchestrated network of interactions between such transcription factors and their associated proteins play a regulatory role during organogenesis and development (Davidson and Erwin, 2006; Raouf et al., 2008). Emerging studies have shown that ER functionally interacts with “pioneer factors” in a coordinated fashion to control the mammary gland cell fate (Jozwik and Carroll, 2012). “Pioneer factors” are transcription factors that can directly bind to condensed chromatin and can bring either positive or negative transcriptional outcomes by recruiting other transcription factors and/or histone modification enzymes (Magnani et al., 2011b; Jozwik and Carroll, 2012). In the recent past, FOXA1, GATA3, PBX1 and AP2gamma are characterized as pioneer factors of ER that play crucial role in decisions regarding fate of cells in mammary gland development (Schuur et al., 2001; Kouros-Mehr et al., 2006; Magnani et al., 2011a).

## FOXA1

FOXA1, a member of the forkhead transcription factor family, participates in pioneer function of the genome regulation (Sekiya et al., 2009). FOXA1 is the first pioneer factor identified for the ER and it co-expresses with ER during mammary gland development, primary breast tumors and cell lines (Carroll et al., 2005; Bernardo et al., 2010). Chromatin binding studies revealed that FOXA1 binds at more than 50% of ER binding sites suggesting that FOXA1 is a key regulator of ER functions in mammary gland development (Hurtado et al., 2011).

FOXA1 is expressed in TEBs, particularly in a subset of luminal cells that are CD61^+^, while it is not expressed in myoepithelial cells (Bernardo et al., 2010). The pattern of expression of FOXA1 matches with ER in the pubertal gland. Because of its critical role in regulation of ER transcription functions and similarity in the expression pattern with ER, KO mouse studies revealed that deficiency of *Foxa1* in mouse leads to defects in hormone-induced mammary ductal invasion associated with a loss of TEB formation (Bernardo et al., 2010). It implies that FOXA1 is essential for the ductal outgrowth during puberty. However, *Foxa1* expression is not required for embryonic development of the mammary ductal rudiment similar to ER. Nevertheless, analysis of mammary glands in *Foxa1* null mice revealed that it is not important for alveologenesis as *Foxa1* null glands could form milk-producing alveoli which are unlikely in *Esr1* null mice. This suggests that *Foxa1* functions are probably restricted to ductal epithelium and independent of alveologenesis. Because of the similarities between the expression pattern and functions in mammary gland, transcriptional interaction between FOXA1 and ER was verified. This analysis indicated that FOXA1 regulates ER expression in luminal cells by directly associating at 10 distinct regions of the *Esr1* gene, with five sites in the promoter region and five in intragenic regions (Bernardo et al., 2010). It implies that FOXA1 contributes to ER functionality and mammary cell fate by modulating the basal expression and functional activity of ER.

## GATA3

GATA3 is a member of GATA transcription factors that participate in development of various organs in the body (Ho and Pai, 2007). Recent reports suggest a pivotal role for GATA3 in mammary gland morphogenesis, particularly in luminal cell differentiation (Kouros-Mehr et al., 2006; Asselin-Labat et al., 2007). GATA3 was identified as highly enriched transcription factor in a microarray screening of mammary epithelium (Kouros-Mehr et al., 2006). During embryonic development, GATA3 expression is detected at E12.5 day in primordial mammary buds (Asselin-Labat et al., 2007) and is confined only to mammary epithelial layer, but not in myoepithelium. Its restricted expression to luminal cell lineages indicates a potential role in the regulation of epithelial cell phenotype of mammary gland development. In consistent with its expression, deletion of *Gata3* results in severe defects in mammary development due to failure in TEB formation during puberty. This phenotype is similar to that observed in loss of ER and it can be further attributed to the failure of the expansion of the luminal progenitor population (Kouros-Mehr et al., 2006). During pregnancy, GATA3 seems to participate in luminal epithelial differentiation required for lobuloalveolar development. The targeted deletion of *Gata3* from progenitor cells blocks luminal cell differentiation, whereas forced expression of GATA3 in mammary stem-cell-enriched populations promotes differentiation into luminal cells. These studies strengthen the fact that GATA3 is required not only to maintain the luminal epithelial lineage, but also in the determination of their cell fate (Asselin-Labat et al., 2007).

**Figure 3:**
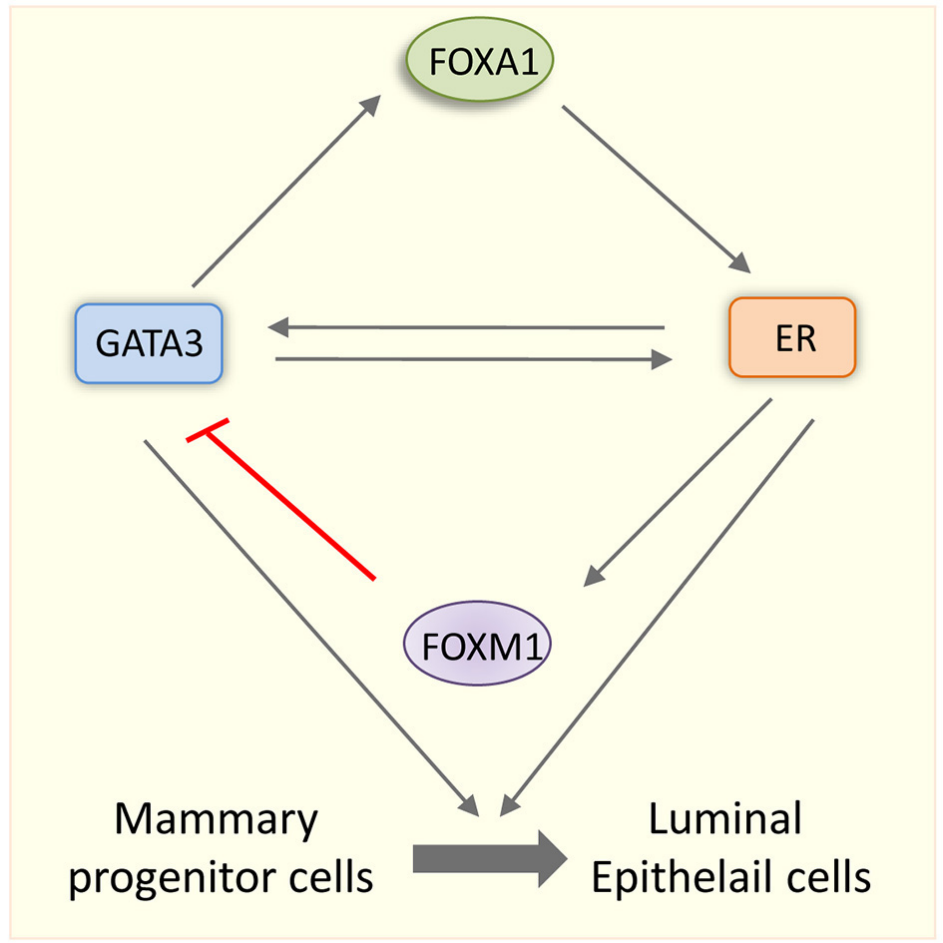
**Interdependence of pioneer factors and ER specifies mammary cell fate**. Schematic representation showing the interplay among ER, GATA3, FOXA1, and FOXM1 in mammary gland cell fate. GATA3 and ER regulate each other's transcription positively. GATA3 also regulate *FOXA1* transcription and FOXA1 in turn upregulates ER in epithelial cells. ER transcriptionally upregulates *FOXM1* and FOXM1 in turn down regulates GATA3. Luminal lineage specific factors such as FOXA1, GATA3, and ER specify the fate mammary progenitor cells and promote their differentiation into luminal epithelial cells.

An intriguing question is that how are GATA3 functions related to ER? Similar to *Esr1* KO mice, conditional deletion of *Gata3* led to the impairment in the TEBs formation suggesting a functional overlap between these two factors. Although GATA-3 and ER pathways may have few non-overlapping functions in mammary luminal cells, FOXA1 bridges the link between ER and GATA3 (Kouros-Mehr et al., 2008). The GATA3 functions are truly integrated into E2 signaling pathway as both of them regulate each other's expression in luminal cells (Kouros-Mehr et al., 2006). GATA3 regulates FOXA1, which in turn mediates ER expression. Further, ER induces GATA3 expression in luminal cells implying that there is an interdependence of FOXA1, ER and GATA3 in the maintenance of luminal cells (Figure 3). Not only that these three transcription factors depend on the transcription of the other, they also colocalize at genomic sites after ligand stimulation results in the formation of a tripartite enhanceosome complex of ERα, FOXA1, and GATA3. This complex further ensures the optimal transcriptional activation by recruiting RNA ploII and p300 to the target gene chromatin (Kong et al., 2011). FOXM1, a forkhead transcription factor, is shown to down regulate GATA3 expression through methylation of the GATA3 promoter in association with DNMT3b, a DNA methyl transferase. Therefore, FOXM1 promotes luminal proliferation by opposing GATA3-mediated luminal differentiation in the mammary gland (Carr et al., 2012). *Foxm1* is an E2 inducible gene (Millour et al., 2010). Therefore, *Foxm1* induction by E2-ER may balance the functional interaction between ER and GATA3 during mammary gland. Taken together, an intricate network of transcription factors involving GATA3, FOXA1, FOXM1, and ER dictates the fate of the mammary cell (Figure 3).

## Concluding remarks

Considering the significant role played by coregulators in ER action, investigation on identification and characterization of new coregulators for ER continues to be an interesting area of research. On the other hand, knowledge on the intricate mechanisms that underlie the coregulator-mediated actions on ER signaling is rapidly expanding. Although, the *in vitro* roles of coregulators on ER functionality and cellular functions are determined, information available on the *in vivo* roles for ER coregulators is limited, considering the large number of known coregulators for ER. This could be due to the fact that complete ablation of the genes of several coregulators led to embryonic lethality. Therefore, in future there is a demand for the utilization of mammary gland-specific conditional KO approach in murine models to address this issue. *In vivo* studies using murine KO models indicated that coregulators like SRC-3, CITED-1 and cyclin D1, and pioneer factors such as FOXA1 and GATA3 are important for the development of pubertal mammary gland. Some coregulators appear to be important in the later stages of mammary gland development, it can be implied that coregulators do not follow a thumb rule in mammary gland development. Balanced expression through interdependency mechanisms among ER, FOXA1, and GATA3 specifies fate of the luminal cell. Considering their established role in mammary gland development and breast cancer, mutant mice models of these coregulators can be further explored for clinical studies in the future. Obtaining the blueprint of all the coregulators and their role in mammary gland development may further offer therapeutic strategies to develop coregulator-based therapies to treat breast cancers.

### Conflict of interest statement

The authors declare that the research was conducted in the absence of any commercial or financial relationships that could be construed as a potential conflict of interest.

## Abbreviations

TEBs: terminal end buds
KO: knockout
Tg mice: transgenic mice
ERα: estrogen receptor alpha
Esr1: estrogen receptor alpha gene 1
SHR: steroid hormone receptor
PTMs: post-translational modifications
PgR: progesterone receptor
MaSC: mammary stem cells.
